# Definitions of Person-Centeredness for Persons With Dementia in the United States: A Scoping Review

**DOI:** 10.1177/07334648261463391

**Published:** 2026-09-08

**Authors:** Lea Efird-Green, London B. Jones, Sam Fazio, Sheryl Zimmerman

**Affiliations:** 1 Cecil G. Sheps Center for Health Services Research and School of Social Work, University of North Carolina at Chapel Hill, Chapel Hill, NC, USA; 2 Alzheimer’s Association Home Office, Chicago, IL, USA

**Keywords:** person-centered, patient-centered, person-directed, definition, Alzheimer’s, dementia, aging, health, scoping

## Abstract

Person-centeredness first emerged as a concept in dementia care in the 1980s. Nearly 40 years later, it has no universal definition, which limits operationalization and implementation of care and supportive services, measures, interventions, policy, and regulation for people with dementia. Thus, a scoping review of the current definitions of person-centeredness in US dementia care was conducted. Of the 71 included studies, there was considerable variability in elements/themes, complexity, and cited theories; the most common themes were needs and preferences, relationships, and personhood, all of which were present in at least half of the studies’ definitions. Importantly, more than 150 articles that were reviewed did not contain a definition, despite including the term in their titles or abstracts. Further investigation in pursuit of a more nuanced conceptualization of person-centeredness that includes these key themes, while also addressing concerns about applicability to diverse cultures and contexts (e.g., organizations and systems), is recommended.

**What This Paper Adds**

• A scoping review that compiles, classifies, and analyzes current definitions of person-centered care for people with dementia in the US.
• The most common elements of person-centeredness for this population are needs and preferences, relationships, and personhood.

**Applications of Study Findings**

• The considerable variability in definitions of person-centeredness in the literature inhibits efforts to operationalize the concept and should be addressed.
• This review provides a foundation on which a nuanced operationalization of the concept of person-centeredness can be built.

## Background

There are more than 7.2 million people living with dementia (PLWD) in the US as of 2025, a number is expected to rise to 12 million by 2040 (Alzheimer’s Association, 2025). All dementias—of which there are several, with Alzheimer’s disease being the most common—are chronic, debilitating, and require support from families and/or health professionals. One of the most common concepts in dementia care is person-centered care. Person-centeredness emerged as a concept first in the field of psychology, with Carl Rogers in the 1940s putting forth the hypothesis that a therapeutic relationship based on “genuineness, nonjudgmental caring, and empathy” would promote a “self-directed grown process” that would improve the mental wellbeing of psychotherapy clients (Raskin and Rogers, 2005, 130). In the 1980s and 1990s, Tom Kitwood used his observations of care delivery to PLWD in long-term care settings to develop the concept of person-centered care as part of a larger social-psychological theory of dementia; this definition focused on the core value of personhood for those living with dementia, which revolutionized the historical medical model that saw dementia strictly as a disease (Kitwood, 1990, 1997).

Since the late 1980s, dementia care has moved from a more clinical treatment model to prioritizing a relationship-based, home-like practice model; Kitwood’s concept of person-centered dementia care contributed significantly to this shift (Love & Kelly, 2011). Person-centered dementia care includes a variety of environmental modifications and care practices such as creating a comfortable non-restrictive physical environment and an emphasis on engagement and decision making for the PLWD (Fazio et al., 2018). These practices are particularly important to encourage non-pharmacological treatment of behavioral expressions of dementia, as well as to improve quality of life throughout the dementia journey (Li and Porock, 2014).

However, as Kitwood and others including Brooker (2004) further elucidated the elements of person-centeredness, differences appeared in the definitions promoted by scholars and providers. For example, Kitwood emphasized the importance of relationships (1997); Brooker stressed creating a positive social environment among other elements (2004); and a 2016 systematic review of the topic found 15 definitions that included 17 principles in six domains (e.g., dignity, choice, and self-determination) (Kogan et al., 2016). A decade ago, Kogan and colleagues argued that this multiplicity of definitions impedes “operationaliz[ation of] a person-centered care approach to health care and services for older adults” and that a consensus definition could benefit the field (2016, e1).

Person-centeredness has also spread to other populations and services, including all aspects of healthcare, further complicating the definition and creating even more terms for the concept (e.g., person-directed care) (Fazio et al., 2018). Given Kitwood’s influence in the dementia care space, person-centered is the most common term in that field, while patient-centered care is more widely used in primary and acute health care, particularly medicine and nursing (Nolte et al., 2020). Patient-centered has a separate history stemming from Balint and other physicians since the 1960s (Institute of Medicine, 2001), while person- or resident-centered care is more common in long-term care, dementia, and aging-related services. Patient-centeredness typically focuses on the clinician-patient interaction and shared decision making for preventive care and treatment of illness (Nolte et al., 2020). There are similar core values across these terms such as the value of relationships, but they are not necessarily synonymous, and none of these terms has a universal definition.

Various reviews of the literature have been published about person- and patient-centeredness, most of which focus on the implementation or effectiveness of interventions, or the application of the concepts to specific fields of healthcare. For example, Lee et al. (2022) reviewed person-centered care interventions for PLWD and conducted a meta-analysis of their effect sizes. And, while there are reviews of definitions of patient-centeredness (Grover et al., 2022; Feldthusen et al., 2022; Sturgiss et al., 2022), none are specific to PLWD or dementia practice; rather, they focus on healthcare as a whole and prioritize the clinician-patient relationship in the context of medicine and/or nursing. In addition, these reviews are not focused on the definitions of patient-centeredness in the US, which affects the specificity of the findings given the unique structure of the US health system (Asadzadeh et al., 2022). Even the previously referenced review by Kogan and colleagues (2016) focused on person-centered care, not definitions of person-centeredness, as well as older adults with chronic conditions and functional impairment rather than PLWD specifically. No other systematic or scoping reviews of definitions of person-centeredness for PLWD in the US exist in the literature.

Therefore, a new scoping review to identify the core concepts of person-centeredness may help advance person-centered policy and practices. Because person-centeredness first emerged from Kitwood’s pioneering philosophy, the dementia care field is uniquely suited to provide common elements for a potential universal definition. This scoping review thus compiled, classified, and analyzed definitions of person-centeredness for persons with dementia drawn from scholarly literature; its research questions are fourfold:

(1) How is the concept “person-centered” and similar terms (person-directed, patient-centered, patient-directed) defined in scholarly literature about persons with dementia in the United States (US)?
(2) What are the similarities and differences among these definitions?
(3) Are there core sub-concepts (e.g., key tenets and common principles) that are widely included in these definitions?
(4) Are there common philosophies or theoretical roots (e.g., Rogers and Kitwood) in these definitions?

## Methods

This scoping review used the widely recognized five-stage Arksey and O’Malley (2005) methodological framework for scoping reviews. This process is similar to the Preferred Reporting Items for Systematic Reviews and Meta-Analyses (PRISMA) systematic review framework, which is used when a review focuses on study outcomes and includes a meta-analysis. The Arksey and O’Malley framework includes “identifying the research question; identifying relevant studies; study selection; charting the data; and collating, summarizing and reporting the results” (2005, 22). Because this is a scoping review, it does not assess the methodological quality or potential for bias in its included literature (Peters et al., 2015) but rather focuses on the presence of a clear definition as the key inclusion criterion.

### Inclusion Criteria

Included literature had to be scholarly and peer-reviewed; be written in English; and be published between 2013 and 2024. Beginning the search in 2013 was decided to ensure that at least 10 years of literature was included in the review, and reflect changes in the dementia care and policy landscapes at that time, especially the creation of the first National Plan to Advance Alzheimer’s Disease in 2012 (US DHHS, 2013) and the redefining of dementia as a major neurocognitive disorder with updated diagnostic criteria in the Diagnostic and Statistical Manual V published in 2013 (Grasset et al., 2018). Additionally, included literature was restricted to research studies and scholarly reviews of the literature to ensure that included articles were more than opinion or commentary; rather, included articles must have used their chosen definition to undergird dementia-focused research. Literature reviews could be systematic, scoping, or otherwise named if they included replicable, systematic search and screening methods. Also, included studies or reviews had to explicitly define “person-centered” or a similar term (person-directed, patient-centered, patient-directed) in the text of the article. These similar terms were used to ensure that dementia-related research in disciplines that traditionally use other terms, including primary and acute care, was included in the review process.

The population was restricted to PLWD regardless of setting. If a study included both PLWD and those without, PLWD had to be purposefully included and used for comparative analyses. If the study participants cared for or worked with PLWD and the study focused on person-centered dementia care, it was also eligible for inclusion. Finally, the data being analyzed in the study or review had to originate in the US due to cultural views on disability and aging, the unique US healthcare system, and resulting dementia care norms (Aranda et al., 2021; Asadzadeh et al., 2022; Ohno et al., 2021). For scholarly reviews, a majority (50% or more) of the studies included in the review must have collected their data in the US.

### Search Process

Four databases were searched as part of this scoping review: PubMed, Cumulative Index to Nursing and Allied Health Literature (CINAHL), American Psychological Association (APA) PsycInfo, and Cambridge Scientific Abstracts (CSA) Social Services Abstracts. Table 1 shows the search fields, filters, and search strings used for each database. Of the 2082 references found, 1052 (51%) came from PubMed; 566 (27%) from CINAHL; 370 (18%) from APA PsycInfo; and 94 (5%) from CSA Social Services Abstracts. The search process was informed by consultation with and assistance from a behavioral and social sciences librarian at the study team’s university. All references were added to Zotero (2023) and formatted for import into Covidence (2023) for deduplication.

**Table 1.** Database Search Fields, Filters, and Strings

Database	Search Field(s)	Filters	Search String
PubMed	Title/Abstract	2013–2024, English	(person-centered [Title/Abstract] OR person-centeredness [Title/Abstract] OR “person centered” [Title/Abstract] OR “person centeredness” [Title/Abstract] OR person-directed [Title/Abstract] OR “person directed” [Title/Abstract] OR patient-centered [Title/Abstract] OR patient-centeredness [Title/Abstract] OR “patient centered” [Title/Abstract] OR “patient centeredness” [Title/Abstract] OR patient-directed [Title/Abstract] OR “patient directed” [Title/Abstract] OR “patient directed” [Title/Abstract]) AND dementia Filters: in the last 10 years, English
CINAHL	Title OR Abstract	Jan 2013–December 2024 (Published Date), English, Peer Reviewed	(person-centered OR person-centeredness OR “person centered” OR “person centeredness” OR person-directed OR “person directed” OR patient-centered OR patient-centeredness OR “patient centered” OR “patient centeredness” OR patient-directed OR “patient directed” OR “patient directed”) AND dementia
APA PsycInfo	Title OR Abstract	Peer Reviewed, 2013–2024 (Publication Year), Exclude Dissertations, English	(person-centered OR person-centeredness OR “person centered” OR “person centeredness” OR person-directed OR “person directed” OR patient-centered OR patient-centeredness OR “patient centered” OR “patient centeredness” OR patient-directed OR “patient directed” OR “patient directed”) AND dementia
CSA Social Services Abstracts	Title OR Abstract	Peer Reviewed, Jan 1 2013–Dec 31 2024, English	(person-centered OR person-centeredness OR “person centered” OR “person centeredness” OR person-directed OR “person directed” OR patient-centered OR patient-centeredness OR “patient centered” OR “patient centeredness” OR patient-directed OR “patient directed” OR “patient directed”) AND dementia

### Screening

**Figure 1.**
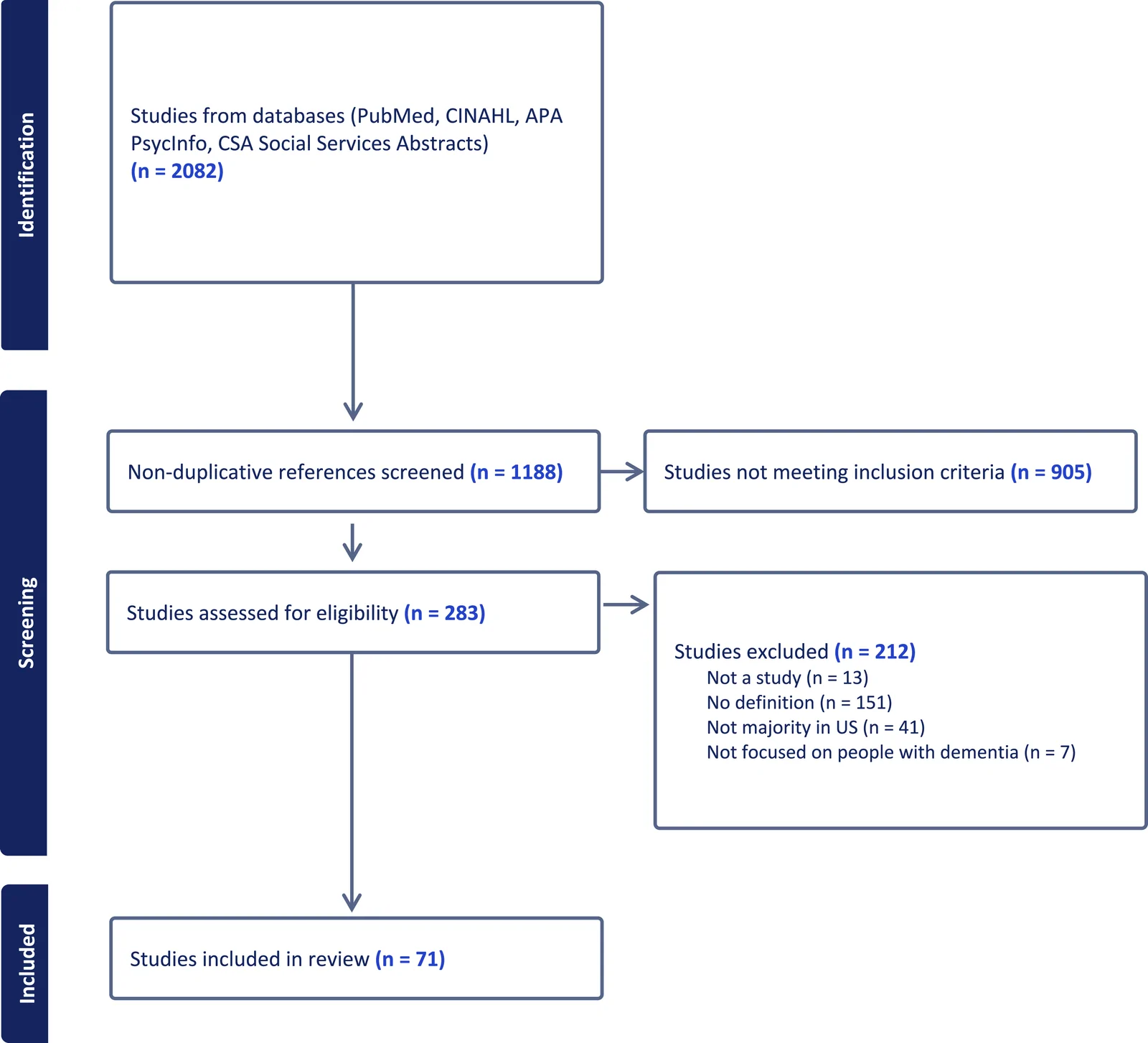
Screening process flow chart

Deduplication of the 2082 references via Covidence (2023) removed 894 references. Covidence (2023) was then used for all screening and extraction processes. Title and abstract screening was completed for 1188 references, and two study team members independently screened each reference to determine if it required full-text review. The initial agreement rate between the two raters was 83%; conflicts were resolved via discussion and subsequent agreement. Of the 1188 references screened, 905 (76%) references were omitted based on the exclusion criteria; the study team then retrieved and reviewed the full text of 283 references. After full-text screening by the same two team members resulted in a 91% agreement rate, the same agreement-based methods were used to exclude 212 (74.9%) references, with 71 references remaining. Full-text exclusion reasons included no definition of term(s) (*n* = 151); majority of data not collected in the US (*n* = 41); not a peer-reviewed study or scholarly review (*n* = 13); and not focused on PLWD (*n* = 7) (see Figure 1). Notably, in all 151 of the references excluded due to lack of a definition, the term “person-centered” or a similar term was used within the title or abstract, highlighting the term’s wide use without an explicit definition. The entire screening process is depicted in Figure 1.

### Extraction and Data Analysis

A data extraction form was created in Covidence (2023) including year published, primary author, article title, journal title, study type, data type, study focus, US state, term, exact definition, page number, and definition reference(s). One team member extracted data from each of the 71 references, which were then checked for accuracy by a second team member within the Covidence platform. Definitions’ references were compiled, and the most common ones reported. These references included Rogers, Kitwood, and national organizations with meaningfully distinct definitions (e.g., Institute of Medicine and Alzheimer’s Association); see Appendix A.

Definition length and specificity were varied; two such examples include “person-centered dementia care honors and respects the unique needs and interests of the person with memory loss (PWML), relying on PWML’s strengths and engaging her/him in activities of interest” (Garlinghouse et al., 2018); and “in person-centered care, the person is central by addressing the whole person as one who has a unique history, interests, preferences, needs, strengths, and abilities. The person-centered approach to dementia care involves: (a) valuing people with dementia; (b) treating them as individuals; (c) understanding the world from the perspective of the person with dementia; and (d) providing a positive social environment where the person’s psychosocial needs for occupation (personally meaningful or purposeful activities), identity, inclusion, attachment, comfort, and agency can be met. The importance of relationships with others in person-centered care is emphasized, through which the intrinsic value of individuals as unique human beings can be nurtured” (Han and Radel, 2017).

Data from the included articles were synthesized using qualitative content analysis (Nelson, 2014), specifically via an iterative inductive coding approach. This approach was chosen so that patterns and themes could emerge directly from the text of each article’s definition (Thomas, 2006) given that deductive coding would instead assume a universal definition or framework of person-centeredness. A codebook was initially drafted by one team member after reviewing each included definition, which was typically one to two sentences long. This codebook initially included six codes based on commonly occurring terms and concepts: needs and preferences, relationships, personhood, beliefs and values, empowerment, and individualization. Each of these codes was operationally described (see “Themes in Definitions of Person-Centeredness”) with examples to enable consistent coding.

This initial codebook was then applied to a subset of definitions by two team members. After discussing the comprehensiveness of the codebook and resolving coding discrepancies through agreement, a final version was developed containing nine codes. This version included the addition of choice and autonomy as a distinct code separate from empowerment; psychological needs, a specific concept of Kitwood’s that is related to personhood but encompasses other terms (e.g., attachment and inclusion); and care environment, which was identified in only one definition. While not as common as the initially identified codes, these additions allowed for a more comprehensive analysis of the data. The codebook was then applied to each definition by one team member and randomly spot-checked by the second team member to ensure accuracy. Ultimately, this process resulted in nine non-mutually exclusive themes of person-centeredness: needs and preferences, relationships, personhood, beliefs and values, empowerment, individualization, choice and autonomy, psychological needs, and care environment.

## Overview of Findings

### Characteristics of Included Studies

There was considerable methodological diversity in this review’s included articles. Of the 71 studies, 13 were mixed methods, 33 were qualitative, and 25 were quantitative. There were 15 case studies; 21 intervention studies; 15 needs assessments; 10 secondary analyses of existing datasets; and 10 systematic/literature reviews. These characteristics are included in Appendix A, which summarizes all included studies’ methodologies and themes. All systematic/literature reviews included a documented thorough search methodology to validate whether their included studies were conducted in the US. The topics of the articles ranged widely and included, among others, staff training and perspectives; family/unpaid caregivers; nutrition; sleep; behavior and symptom management; psychosocial wellbeing; dementia in both residential- and community-dwelling individuals; and the cultural connotations of dementia and caregiving across racial and ethnic groups. Of the 71 included studies, 68 used the term “person-centered,” 2 used “patient-centered,” and 1 used “person-directed.”

### Themes in Definitions of Person-Centeredness

Many definitions (90%) included more than one theme; all themes and their frequencies are shown in Table 2. The most common theme was needs and preferences, which was included in 42 (59%) of the definitions. Needs and preferences of PLWD included multiple types of needs—for example, “the psychosocial and physical needs of PLWD” (Osakwe et al., 2021), or “meet both their [PLWDs’] needs and abilities” (Tak, 2021)—along with preferences, often used verbatim such as in “resident… preferences and interests” (Sefcik et al., 2020). This theme also encompassed holistic statements about the needs and capacities of PLWD such as “meeting residents where they are” (Travers et al., 2022).

**Table 2.** Frequency of Themes in 71 Articles

Theme	Number of Definitions	Percent of Definitions
Needs and Preferences	42	59%
Relationships	37	52%
Personhood	34	48%
Beliefs and Values	22	31%
Empowerment	16	23%
Individualization	15	21%
Choice and Autonomy	11	15%
Psychological Needs	6	8%
Care Environment	1	1%

The second most common theme, present in 37 (52%) of the definitions, was relationships, which included relationships among care partners (paid and/or unpaid) and PLWD as well as the social environment (e.g., relationships among peers and other social interactions). In addition, concepts such as “shared decision-making” (Nowaskie et al., 2020; Osakwe et al., 2021) require relationships among caregivers and PLWD and their families and as such were included in this theme. Relationships were integral to the ability of others to understand the perspectives and needs of the person with dementia; for example, “knowing the individual through interpersonal relationships” (Liu et al., 2022); “prioritizing relationships to the same extent as care tasks” (Lopez et al., 2013); and “formation of rewarding relationships through conversation and caregiving” (Savundranayagam, 2014) were identified as key factors in providing dementia care.

The next most frequent theme was personhood, present in 34 (48%) of definitions and defined by Kitwood as “a standing or status that is bestowed upon one human being, by others, in the context of relationship and social being… it implies recognition, respect, and trust” (Sauer et al., 2016). Personhood was both explicitly named as a term and implicated through other terms such as “being worthy of respect and dignity” (Staehler et al., 2022) or “entering [PLWDs] world and assuming that there is meaning in all behavior” (Gaugler et al., 2013). Beliefs and values was a theme in 22 (31%) of the included articles’ definitions. In these definitions, person-centered dementia care had to recognize the “rights, values, and beliefs of the individual [with dementia]” (Gaugler et al., 2015). Both beliefs and values were often explicitly named, although other ways of describing the theme, such as “biographical, cultural, and social aspects of the person with dementia” (Doyle and Rubinstein, 2014) or “unique set of… views [and] histories” (Kolanowski et al., 2015), were also present.

The theme of empowerment was present in 16 (23%) definitions, which included the specific term “empower” as well as other power-sharing concepts such as “shared decision-making” (Gaugler et al., 2015) and “supporting/enabling a person” (Calkins et al., 2022). The final theme present in at least 20% of the definitions was individualization (15; 21%). This theme was more specific than the needs and preferences theme, in that it used the terms “individualize/d” or “personalize/d,” for example, “individualized and reflective of focusing on the person rather than the care task” (Gilmore-Bykovskyi and Rogus-Pulia, 2018).

Less common themes included choice and autonomy (11 definitions; 15%); Kitwood’s psychological needs (6 definitions; 8%); and care environment (1 definition; 1%). Choice and autonomy had to specifically include one of those terms or a synonym—such as “maximizing choice and autonomy” (Hao and Ruggiano, 2020)—and differ from the concept of empowerment that implies power-sharing. Kitwood’s psychological needs of people living with dementia include “safe-guarding the identity, inclusion, attachment, comfort, and occupation of individuals” (Li and Porock, 2014) and is a concept specifically focused on personhood. Care environment was unique in that only one study included that theme, largely because no other themes in this review were applicable; in full, that definition was “person-centered care is based on the knowledge that the care environment plays a large role in determining the nature of the experience and the course of the disease” (Davis and Shenk, 2015).

Included articles not only had disparate definitions, but also disparate levels of complexity; that is, some definitions included multiple themes (four to five) versus only one or two themes. Additionally, some authors had multiple publications between 2013 and 2024 that included different definitions of person-centeredness; this was the case for Savunndranayagam and team in (2014, 2015, and 2016), and for six other first authors. There were no noticeable trends in definition themes over time (e.g., earlier vs. more recent articles), in methodology (data type, study/review type), or in intervention topic (e.g., staffing, caregiver perceptions). For example, case studies’ definitions did not contain a single common theme, nor an exclusive theme not present in other methodologies’ definitions. Definitional consistency was therefore variable both within and among research teams, intervention foci, methodology, and over time.

### References of Included Definitions

The sources of the included studies’ definitions were compiled to analyze use of common references or philosophies. Most included definitions had a single theory referenced, but 8 (11%) had two primary references. A majority of included studies referenced Kitwood; 22 (31%) referenced his work directly, and another 16 (23%) referenced a work that itself cited Kitwood when defining person-centeredness (denoted as “2nd level” in Appendix A). A total of 6 (8%) cited Carl Rogers, and 22 (31%) used organizational definitions, including 11 (15%) from the Alzheimer’s Association, 8 (11%) from the Institute of Medicine, 3 (4%) from the Centers for Medicare & Medicaid Services (CMS), and 1 (1%) from the Patient-Centered Outcomes Research Institute (PCORI). A total of 8 (11%) did not reference another work, and 4 (6%) referenced a work that did not directly reference any of the above sources.

## Discussion, Implications, Limitations, and Conclusion

### Discussion

Person-centeredness is a complex term that is common in dementia-related research but has no consistent definition, which impedes its use in practice and policy (Forsgren et al., 2025; Corazzini et al., 2019). This scoping review of definitions of person-centeredness for persons with dementia in the US found 71 articles that met inclusion criteria and defined person-centered or similar terms (e.g., patient-centered and person-directed). During the review process, more than 150 articles that met other inclusion criteria did not contain a definition of person-centered or a similar term, despite having included it in their titles and/or abstracts. Many of these excluded articles specifically tested person-centered interventions or conducted needs assessments in pursuit of person-centeredness but did not define what the term meant in context. This observation is concerning given that there is no agreed-upon definition of person-centeredness (Behrens et al., 2019), indicating that contextual definitions are required to determine which elements of the concept are being used as a theoretical basis for the intervention of interest. Even if the term is defined, however, the person-centeredness of a potential practice or policy is difficult to assess accurately because measures of person-centeredness are not conceptually uniform across settings and populations, thereby complicating the implementation and evaluation of person-centered practice and policy (Corazzini et al., 2019; Heid et al., 2024).

In this review, nine non-mutually exclusive themes were identified that defined person-centeredness and similar terms, the most common being needs and preferences, relationships, and personhood, all of which were present in half or more of the definitions. Of the nine themes, six were present in at least 20% of definitions, although there was significant variety in both the themes present and the complexity of the definitions themselves (i.e., the number of themes in a single definition). Most definitions included more than one theme (90%), but in several instances a single definition included up to five themes. Also, the themes ranged in definitional and implementational complexity. For example, personhood is a psychological theory that is hard to define, while choice and autonomy are simpler to define but difficult to implement due to issues of safety and wellbeing.

Importantly, multiple publications by the same authors did not include the same themes in their definitions of person-centeredness. If consistency of definition cannot be guaranteed within a research team, it is unlikely that consistency among disparate researchers over time is possible without standardization. In addition, there were several underpinning philosophies cited in the included articles’ definitions of person-centeredness. However, Kitwood (1997), who first proposed the idea of person-centered dementia care, was by far the most common, with 54% of included studies citing Kitwood directly or a work that itself cited Kitwood. The preponderance of references to Kitwood over 25 years after he first proposed the concept is a testament to its staying power. However, those definitions referencing Kitwood did not consistently include the same themes, which calls into question the operationalization of his concept in dementia care, let alone across other populations and settings.

The most common themes found in this review are widely acknowledged as integral to person-centered care delivery for PLWD and other populations. For example, the Centers for Medicare & Medicaid Services (CMS) includes, among other concepts, preferences and provider-patient communication (i.e., relationships) in their definition of person-centered care, which drives healthcare regulations and reimbursement models (CMS, 2023). The Alzheimer’s Association’s Dementia Care Provider Roundtable similarly identified tailoring care to the needs and preferences of PLWD and making relationships foundational to create a person-centered culture (Fazio et al., 2020). Preferences are particularly of interest to care providers as they can improve personalized care that is meaningful to PLWD and others (Abbott et al., 2018). However, developing true relationships with PLWD and meeting their needs and preferences is complex, in part due to the inherent tensions in person-centered care, for example, safety versus freedom (Zimmerman, 2026). More complex themes, such as personhood and empowerment, are especially difficult to implement; for example, can a PLWD be truly empowered when they are limited in their ability to make decisions?

## Implications

The difficulty in providing personalized dementia care, let alone truly person-centered care that requires a culture shift that values the inherent worth of people living with dementia (Kitwood, 1997), calls into question the feasibility of universally implementing person-centered care without a common understanding of person-centeredness. In addition, the included articles primarily focused on the provision of person-centered dementia care, rather than the creation of person-centered organizations or systems, which are required for the true implementation of person-centered care (Behrens et al., 2019; Corazzini et al., 2019). Without supportive systems, person-centered care cannot be integrated across health settings (Heid et al., 2024; Kogan et al., 2016), meaning that person-centeredness may require a more expansive definition that is applicable to larger societal structures as well as an individual’s care and quality of life (Efird-Green, 2026). While there are large-scale efforts to improve clinical and healthcare practices for older adults and PLWD such as the 4M Framework (Mate et al., 2021) and the Alzheimer’s Association Dementia Care Practice Recommendations (Fazio et al., 2020), these frameworks do not necessarily encapsulate all aspects of person-centeredness and primarily focus on the individual’s experience rather than a broader perspective such as addressing resource disparities.

Even if the included definitions covered every aspect of person-centeredness, merging them into one definition would fall short because true person-centeredness extends beyond interpersonal interactions to communities, organizations, and systems to best support the wellbeing of people with dementia. Also, a monolithic definition would not account for different cultural understandings of person-centeredness, which is an inherently Western and humanistic concept that focuses on the individual and is not universally applicable or ethical to collectivistic cultures (i.e., those that focus on the good of the group) (Arnold et al., 2020). Therefore, a more nuanced definition of person-centeredness that integrates its philosophical roots, including themes from the literature, while being adequately expansive so as to be applied on any socioecological level and in a variety of settings, populations, and cultural contexts, is necessary. This nuanced definition could function as a conceptualizing framework addressing the relevance of person-centeredness to different contexts while still supporting operationalization, implementation, and more standardized measurement. To have a foundational basis in the literature, this conceptualization should include the key themes—most notably needs and preferences, relationships, and personhood—all of which were present in half of this review’s included definitions.

In addition to a lack of a universal definition or framework of person-centeredness, there is a dearth of validated measures to evaluate the effectiveness of person-centered interventions that are in and of themselves person-centered (Ebrahimi et al., 2021; Molony et al., 2025). For example, most measures that evaluate quality of life for PLWD are not person-centered or strengths-based; rather, they assess physical and cognitive decline, including the inability to complete activities of daily living (Clarke et al., 2020; Molony et al., 2023). The person-centered measures that do exist have disparate operationalizations of the concept, stemming from the lack of a universal definition (Kogan et al., 2016; Edvardsson and Innes, 2010). Therefore, there is not only need for a new conceptualization of person-centeredness, but also measures that are truly person-centered in both design and execution and incorporate the key themes found in this review. Recommendation includes measures that focus on the quality of life of PLWD that incorporate personhood, relationships, needs, and preferences; that prioritize the perspective of the PLWD such as by including them in measurement development; use of self- rather than proxy-reporting; and minimizing cognitively taxing language and answer formats (Mast et al., 2021; Molony et al., 2025; Zimmerman, et al., 2026). Such measures could serve as a clinical tool for care improvement as well as a research tool to evaluate person-centered interventions.

### Limitations

The potential limitations of this review are in its search strategy and inclusion criteria. It is possible that there were definitions that were missed if the search terms did not capture certain articles, or articles were not present in any of the searched databases or journals. By the nature of its inclusion criteria, the results of this review are particular to dementia-related care and practice and to the US and may not be universally applicable in other contexts and cultures. In addition, literature that did not include data (e.g., editorials and commentary) and gray literature were not included in this review due to its focus on defining person-centeredness in scholarly literature.

## Conclusion

This scoping review searched and assessed the recent scholarly literature to determine how person-centeredness is defined for PLWD in the US. Results indicate that many studies do not define person-centeredness (or similar terms) even when using the term in their titles and/or abstracts, and that when it is defined, there are a variety of thematic elements present. Complexity and theoretical underpinnings also differed across included definitions. This inconsistency impairs operationalization and implementation of person-centeredness for PLWD. A more nuanced conceptualization of person-centeredness that includes the key themes—needs and preferences, relationships, and personhood—is recommended to address concerns about discrepancies in definitions while supporting the application of person-centeredness to both individual care and relationships as well as the organizations and systems that support the wellbeing of people with dementia.

## Supplemental Material

Supplemental Material - Definitions of Person-Centeredness for Persons With Dementia in the United States: A Scoping Review Supplemental Material for Definitions of Person-Centeredness for Persons With Dementia in the United States: A Scoping Review by Lea Efird-Green, London B. Jones, Sam Fazio, and Sheryl Zimmerman in Journal of Applied Gerontology
