# Supplementary material for: Definitions of Person-Centeredness for Persons With Dementia in the United States: A Scoping Review
Source: J Appl Gerontol. 2026 Sep 8;45(1 Suppl):17S–29S. doi: 10.1177/07334648261463391 (PMC13556327; doi:10.1177/07334648261463391)
Supplement: Supplementary file 1 [file sj-pdf-1-jag-10.1177_07334648261463391.pdf]

Appendix A: Methodological Characteristics and Definition Themes of Included Studies

Study	Study Type	Data Type	Definition Themes	References
Albaroudi 2022	Secondary Analysis	Quantitative	needs and preferences; relationships	None
AllenChristensen 2019	Systematic Review	Qualitative	personhood; beliefs and values; relationships; empowerment; choice and autonomy	Other
Armstrong 2021	Needs Assessment	Qualitative	needs and preferences; beliefs and values	Institute of Medicine
Barrios 2016	Needs Assessment	Mixed-Methods	needs and preferences; relationships	Patient-Centered Outcomes Research Institute
Boafo 2023	Case Study	Qualitative	personhood; relationships; psychological needs (Kitwood)	Kitwood
Brown 2020	Systematic Review	Quantitative	relationships; needs and preferences	Alzheimer's Association
Burshnic 2018	Needs Assessment	Quantitative	personhood; relationships; empowerment; needs and preferences	Rogers (2nd Level) [a]; Kitwood (2nd Level) [a]
Calkins 2022	Systematic Review	Quantitative	personhood; relationships; beliefs and values; needs and preferences; empowerment	Rogers; Kitwood
Campbell 2024	Secondary Analysis	Qualitative	needs and preferences; beliefs and values	Kitwood; Alzheimer's Association
Davis 2015	Intervention Study	Mixed-Methods	care environment	None
Douglas 2022	Case Study	Quantitative	choice and autonomy	Kitwood (2nd level) [a]
Douglas 2024	Needs Assessment	Qualitative	needs and preferences	Other
Doyle 2014	Case Study	Qualitative	personhood; relationships; beliefs and values; individualization	Kitwood
Fazio 2018	Other Review (Narrative, Literature)	Qualitative	relationships; needs and preferences	Rogers; Kitwood
Garlinghouse 2018	Intervention Study	Mixed-Methods	needs and preferences; beliefs and values	None
Gaugler 2013	Intervention Study	Quantitative	beliefs and values; empowerment; personhood; relationships	Kitwood (2nd level) [a]
Gaugler 2015	Intervention Study	Mixed-Methods	beliefs and values; empowerment; personhood; relationships	Kitwood (2nd level) [a]
Gilmore-Bykovskyi 2015	Case Study	Quantitative	individualization	Kitwood (2nd level) [a]

Gilmore-Bykovskyi 2018	Secondary Analysis	Quantitative	individualization	Kitwood (2nd level) [a]
Han 2016	Intervention Study	Qualitative	needs and preferences; personhood	Kitwood (2nd level) [a]
Han 2017	Intervention Study	Qualitative	needs and preferences; personhood; psychological needs (Kitwood); relationships	Kitwood
Han 2020	Case Study	Qualitative	beliefs and values; personhood	None
Hao 2020	Systematic Review	Quantitative	personhood; relationships; needs and preferences; beliefs and values; choice and autonomy	Rogers; Kitwood (2nd level) [a]
Heid 2024	Intervention Study	Quantitative	needs and preferences; personhood	Institute of Medicine (2nd level) [a]
Hrybyk 2021	Case Study	Qualitative	personhood; individualization	Kitwood (2nd level) [a]
Ihara 2019	Intervention Study	Quantitative	beliefs and values; empowerment; relationships	Kitwood
Inoue 2021	Case Study	Qualitative	needs and preferences; beliefs and values	Institute of Medicine
Kasler 2024	Intervention Study	Mixed-Methods	needs and preferences; beliefs and values; individualization	Institute of Medicine (2nd level) [a]
Kemp 2024	Intervention Study	Qualitative	needs and preferences; personhood	Centers for Medicare and Medicaid Services
Kennedy 2024	Intervention Study	Mixed-Methods	needs and preferences	Kitwood
Kolanowski 2015	Needs Assessment	Qualitative	choice and autonomy; personhood; beliefs and values	Institute of Medicine (2nd level) [a]
Li 2014	Other Review (Narrative, Literature)	Mixed-Methods	personhood; psychological needs (Kitwood); relationships	Rogers; Kitwood
Li 2017	Intervention Study	Quantitative	personhood; relationships; empowerment; psychological needs (Kitwood)	Kitwood
Liu 2017	Secondary Analysis	Quantitative	needs and preferences; choice and autonomy; empowerment; relationships	Other
Liu 2022	Case Study	Mixed-Methods	needs and preferences; relationships	Alzheimer's Association
Liu 2022	Case Study	Mixed-Methods	needs and preferences; individualization	Alzheimer's Association
Liu 2023	Secondary Analysis	Mixed-Methods	individualization; needs and preferences	Alzheimer's Association
Liu 2023	Secondary Analysis	Mixed-Methods	personhood; needs and preferences; relationships	Kitwood (2nd level) [a]
Long 2022	Intervention Study	Quantitative	needs and preferences; beliefs and values	Kitwood (2nd level) [a]

Lopez 2013	Needs Assessment	Qualitative	personhood; relationships; empowerment	Kitwood
Madrigal 2022	Secondary Analysis	Quantitative	needs and preferences; beliefs and values	Alzheimer's Association
Mast 2021	Systematic Review	Quantitative	beliefs and values; choice and autonomy; relationships; individualization	Kitwood
McClendon 2013	Needs Assessment	Quantitative	personhood; choice and autonomy	Kitwood
Meyer 2022	Intervention Study	Qualitative	needs and preferences; relationships; personhood	Alzheimer's Association
Molony 2023	Case/Cohort Study	Qualitative	personhood; relationships	Kitwood
Nowaskie 2020	Needs Assessment	Mixed-Methods	relationships; empowerment	Kitwood
Osakwe 2021	Needs Assessment	Quantitative	personhood; needs and preferences; relationships; empowerment; individualization	Kitwood; Alzheimer's Association
Pleasant 2017	Intervention Study	Quantitative	relationships; empowerment	Kitwood (2nd level) [a]
Porock 2015	Needs Assessment	Qualitative	beliefs and values	Institute of Medicine
Reckrey 2024	Needs Assessment	Qualitative	needs and preferences; relationships; individualization	Alzheimer's Association
Redford 2023	Intervention Study	Quantitative	needs and preferences; choice and autonomy	Alzheimer's Association
Reinhardt 2020	Intervention Study	Quantitative	needs and preferences; relationships	None
Resnick 2018	Needs Assessment	Quantitative	choice and autonomy; personhood; needs and preferences	Other
Resnick 2021	Intervention Study	Quantitative	needs and preferences	Centers for Medicare and Medicaid Services
Sadarangani 2021	Needs Assessment	Qualitative	needs and preferences; relationships	None
Sauer 2016	Intervention Study	Qualitative	personhood; psychological needs (Kitwood)	Kitwood
Savundranayagam 2014	Case Study	Qualitative	beliefs and values; needs and preferences; relationships	Kitwood
Savundranayagam 2015	Case Study	Qualitative	personhood; beliefs and values; needs and preferences; relationships; psychological needs (Kitwood)	Kitwood
Savundranayagam 2016	Case Study	Qualitative	personhood; empowerment; relationships; individualization	Kitwood (2nd level) [a]
Sease 2024	Needs Assessment	Qualitative	needs and preferences; choice and autonomy; personhood; relationships	Alzheimer's Association

Sefcik 2020	Case Study	Quantitative	beliefs and values; needs and preferences	Centers for Medicare and Medicaid Services
Serbser-Koal 2024	Other Review (Narrative, Literature)	Qualitative	personhood; relationships	Kitwood
Staehler 2022	Secondary Analysis	Qualitative	personhood; beliefs and values	Rogers; Kitwood
Tak 2015	Intervention Study	Mixed-Methods	personhood; individualization	Kitwood (2nd level) [a]
Tak 2021	Other Review (Narrative, Literature)	Qualitative	personhood; empowerment	None
Tompkins 2020	Intervention Study	Quantitative	personhood; care delivery; choice and autonomy	Kitwood (2nd level) [a]
Travers 2022	Needs Assessment	Qualitative	needs and preferences; relationships; empowerment; individualization	None
Wang 2024	Scoping Review	Qualitative	needs and preferences; relationships; empowerment	Institute of Medicine (2nd level) [a]
Williams 2018	Secondary Analysis	Qualitative	beliefs and values; personhood; needs and preferences	Kitwood
Xu 2024	Secondary Analysis	Qualitative	personhood; individualization; relationships	Kitwood (2nd level) [a]
Yevchak 2017	Case Study	Qualitative	needs and preferences; individualization	Institute of Medicine (2nd level) [a]

[a] "2nd level" indicates that the cited reference in the included article in turn cites the listed reference. For example, Kitwood (2nd level) indicates that the included article (Gaugler 2013) cited another article (Edvardsson 2008) that itself cited Kitwood in its definition of person-centeredness.
